# Computed Tomographic Tenography of Normal Equine Digital Flexor Tendon Sheath: An Ex Vivo Study

**DOI:** 10.1155/2015/903169

**Published:** 2015-06-22

**Authors:** Luca Lacitignola, Pasquale De Luca, Alessandro Guarracino, Antonio Crovace

**Affiliations:** Sezione di Cliniche Veterinarie e P.A., Dipartimento delle Emergenze e Trapianti di Organo, Università degli Studi di Bari “Aldo Moro”, s.p. per Casamassima km 3, Valenzano, 70010 Bari, Italy

## Abstract

Aim of this study was to document the normal computed tomographic tenography findings of digital flexor tendon sheath. Six ex vivo normal equine forelimbs were used. An axial approach was used to inject 185 mg/mL of iopamidol in a total volume of 60 mL into the digital flexor tendon sheaths. Single-slice helical scans, with 5 mm thickness, spaced every 3 mm, for a pitch of 0.6, and with bone algorithm reconstruction, were performed before and after injections of contrast medium. To obtain better image quality for multiplanar reconstruction and 3D reformatting, postprocessing retroreconstruction was performed to reduce the images to submillimetre thickness. Computed tomographic tenography of digital flexor tendon sheaths could visualize the following main tendon structures for every forelimb in contrast-enhanced images as low densities surrounded by high densities: superficial digital flexor tendon, deep digital flexor tendon, manica flexoria, mesotendons, and synovial recess. Results of this study suggest that computed tomographic tenography can be used with accuracy and sensitivity to evaluate the common disorders of the equine digital flexor tendon sheath and the intrathecal structures.

## 1. Introduction

Equine digital flexor tendon sheath (DFTS) lesions are usually investigated using radiography, ultrasonography, and tenoscopy; contrastography has also been successfully employed to detect pathologic conditions involving the DFTS of sport horses [1]. Normal radiographic anatomy of the DFTS using contrast tenography for the investigation of chronic tenosynovitis was described in 1986 [2, 3].

Deep digital flexor tendon (DDFT) or manica flexoria (MF) tears have been demonstrated to be the most frequently associated lesions of nonseptic tenosynovitis of digital tendon sheath in the horse [1, 3, 4].

Ultrasonography and contrast radiography of DFTS have been employed as principal diagnostic techniques, with different reported sensitivity [1, 3, 4]. The sensitivity of ultrasonography in diagnosing MF tears has been shown to be poor. In a retrospective analysis of 76 cases of nonseptic tenosynovitis of DFTS, the tears of the MF were predicted with a sensitivity of 38% compared with DDFT tears, which were predicted with a sensitivity of 71% [4]. Contrast radiography was found to delineate the border of the MF accurately and, with a description of the normal orientation of the MF, evaluators were able to identify an abnormal appearance and therefore diagnose MF tears with a sensitivity which exceeds that of ultrasonography [1]. 

However, advances in ultrasound technology and operator experience led to a decline in the use of contrast radiography before these injuries were first recognised upon the advent of tenoscopy [1, 5].

Although computed tomography (CT) has been increasingly used in some veterinary institutions to evaluate the equine appendicular skeleton, including the stifle joint, carpus, hock, and fetlock [6–10], unfortunately, visualization of the soft tissues by CT is limited and commonly unrewarding without the use of intravascular or intrasynovial contrast.

Computed tomographic arthrography (CTR) was recently described as an imaging technique that uses intra-articular administration of contrast to improve visualization of the intrasynovial and perisynovial soft tissues [7, 9]. The contrast medium diffuses within the joint compartments, resulting in more conspicuous structures. The soft tissue structures appear as filling defects outlined by the contrast medium in the joint. CTR has been used to identify abnormalities of the articular surfaces, intrasynovial soft tissue structures, the synovial surface of joint capsules, and periarticular structures closely associated with the joint compartments.

At the time of writing, there were no published descriptions of DFTS tenography performed with the use of CT. The aim of this study was to determine the normal findings of DFTS using computed tomographic tenography on the ex vivo limbs of horses.

## 2. Material and Methods

### 2.1. Specimens

A cadaveric study was performed to evaluate a total of 6 equine distal forelimbs, collected at the slaughterhouse, from horses of unknown breed and age. The limbs were amputated at the level of the carpometacarpal joint and were examined to exclude limbs with any gross anatomical abnormalities or damage produced during the cutting process. The specimens were used fresh and the hair was clipped. The limbs were positioned with the palmar surface on the table, and one specimen was also scanned in lateral recumbency.

### 2.2. Contrast Medium (CM) Injection

Injection of DFTS was performed using the axial approach. With the metacarpophalangeal joint flexed to a 225° angle, the needle was inserted at the level of the midbody of the lateral proximal sesamoid bone, through the palmar annular ligament, and 3 mm axial to the palpable palmar or plantar border of the lateral proximal sesamoid bone, in the transverse plane, and directed 45° from the sagittal plane, angled toward the central intersesamoidean region, approximately 15 to 20 mm in depth [11]. The contrast medium iopamidol, 370 mg/mL (IOPAMIRO 370, Bracco; Rome, Italy), was diluted 1 : 1 with saline solution (0.9%) to a final concentration of 185 mg/mL, and a total of 60 mL was injected, except for 1 specimen that was injected with 30 mL to test the grade of synovial distension.

### 2.3. CT Scanning

A third generation single-slice CT scanner (GE Prospeed Power SX, GE Healthcare, Milwaukee, WI) was used for all procedures. The helical scan mode was used with the following parameters: 120 kVp, 130 mA, 1.0 sec scan time, 5 mm slice thickness, and 5 mm table advancement. The standard acquisition preset of a 25 cm field of view, matrix of 512 × 512 pixels, bone reconstruction algorithm, and WL of 300 and WW of 1000 was used. The raw dataset was retroreconstructed to a slice thickness of 0.3 mm. To obtain better reconstruction images, the overlapping technique, 5 mm slice thickness and 3 mm table advancement, pitch 0.6, (X-ray beam in overlapping) was also performed.

Images were visualized using PACS software (OsiriX DICOM Viewer, Pixmeo; Bernex, Switzerland), for transverse scanning, multiplanar reformatting (MPR), and 3D-rendering reconstruction.

CT images of each limb were analysed by 3 expert clinicians (L. L., A. G., and P. D. L.) and 2 students in veterinary medicine using the same software. An anatomical textbook [12] was used as reference for identifying normal anatomical structures in the CT tenography images. Each observer described the following normal structures for each specimen examined: in transverse scans, the proximal pouch of the DFTS synovial cavity, palmar aspect of the DFTS synovial cavity, synovial collateral recess, distal synovial fold, dorsal distal recess of the DFTS, superficial digital flexor tendon (SDFT), MF, DDFT, mesotendon, intersesamoidean ligament, straight sesamoidean ligament, and mesotendons; in MPR and 3D reformatted images, the dorsal proximal recess, palmar proximal recess, collateral recess, palmar distal recess, and dorsal distal recess. The observer assigned a score for each structure as follows: 0 if the structure was not identifiable, 1 if it is difficult to identify, and 2 if it is easy to identify; the total maximum score obtainable for a specimen was 34. The presence of artefacts (air bubbles, contrast medium leakage, reinforced beam, and inadequate distension) was also assigned a score as follows: 0 not appreciable, 1 slightly appreciable, and 2 highly appreciable.

### 2.4. Statistical Analysis

Data were analysed using Minitab 15.1 Statistical Software (Minitab, Inc.; State College, PA, USA). Data was analysed for normal distribution and analysis of variance (ANOVA) was performed to compare the scores assigned to the structures and artefacts by the expert versus the student observers and to compare the scores of the specimens. The significance level was set at *p* < 0.05.

## 3. Results

### 3.1. Transverse Scans

High-quality transverse scan images were obtained using either the contiguous or the overlapping scanning technique. The volume of CM used was adequate and provided intrathecal distension sufficient for separating anatomical structures and facilitating interpretation of the images. Intrathecal digital tendon structures were visualized at low density ranging from 150 to 240 Hounsfield Units (HU), and the synovial cavity was visualized with CM (>2000 HU). The MFs were also visible, especially if there was a high pressure of CM. Some artefacts were due to CM leakage that penetrated the subcutaneous tissue surrounding the point of injection and air bubbles that accumulated in the proximal DFTS pouch. In the specimen injected with 30 mL of CM solution, no leakage was verified, but less synovial distension was obtained, leading to difficulty in identifying the MF. Limb positioning (lateral or palmar recumbency) did not affect the distribution of CM in the synovial cavity; however, air bubbles were found in the uppermost regions of the cavity.

**Figure 1:**
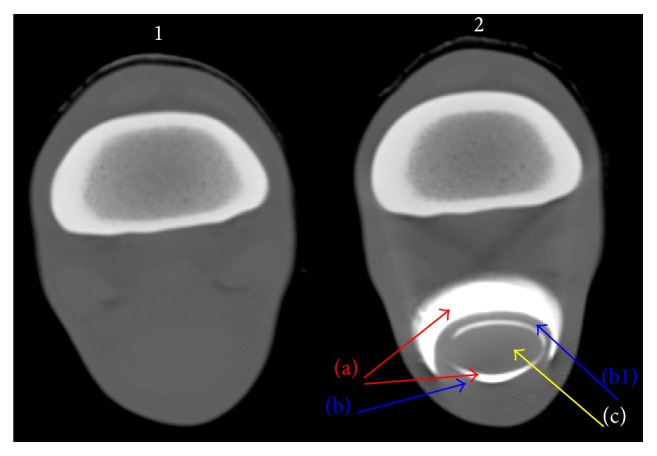
Normal representative images of transverse scans of direct CT (scan 1), and CT tenography (scan 2) of ex vivo forelimb of horses. (a) Proximal pouch of DFTS synovial cavity, (b) SDFT, (b1) manica flexoria, and (c) DDFT.

**Figure 2:**
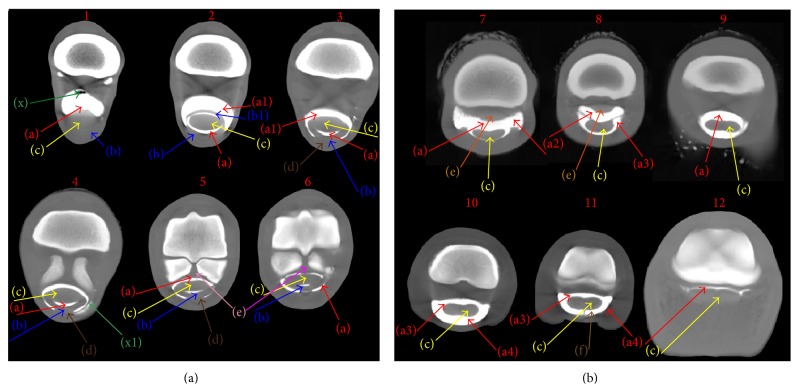
Computed tomographic tenography of ex vivo normal forelimbs of horses: representative images of transverse scans. Scans are shown proximally to distally: scans 1 to 6 of metacarpal region and 7 to 12 of pastern region. (a) Proximal pouch of digital flexor tendon sheath (DFTS) synovial cavity, (a1) palmar aspect of DFTS synovial cavity, (a2) synovial collateral recess, (a3) distal synovial fold, (a4) dorsal distal recess of DFTS, (b) superficial digital flexor tendon (SDFT), (b1) manica flexoria, (c) deep digital flexor tendon (DDFT), (d) mesotendon, (e) intersesamoidean ligament, (f) straight sesamoidean ligament, (g) mesotendon, (x) artefact and air bubbles, and (x1) subcutaneous contrast medium (CM) outflow after injection.

Figure 1 shows representative images obtained from the transverse scans. The intrathecal regions of the main tendon structures such as SDFT and DDFT were easy to identify, whereas the extrathecal regions were difficult to appreciate. High pressure of CM into the synovial cavity adequately separated the MF from the DDFT and synovial membrane, allowing accurate identification (Figures 2(a) and 2(b)). Transverse scans performed at the proximal sesamoid bones level showed a low volume of CM in the synovial cavity, whereas, in the pastern region, the distal DFTS pouch and synovial folds were clearly identified, because there was adequate distension by CM. The mesotendon was easily identified at this level in every specimen (Figure 2(b), scan 11, letter f).

### 3.2. MPR and 3D Reconstruction

**Figure 3:**
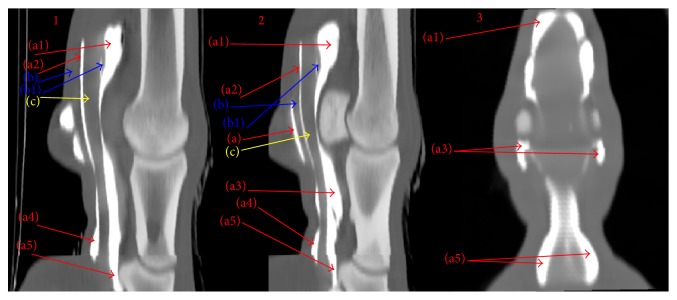
Multiplanar reconstruction (MPR). Computed tomographic tenography of ex vivo normal forelimbs of horses: representative images of sagittal (1), parasagittal (2), and dorsal (3) scans of DFTS. (a1) Dorsal proximal recess, (a2) palmar proximal recess, (a3) collateral recess, (a4) palmar distal recess, and (a5) dorsal distal recess. (b) SDFT and (b1) manica flexoria.

**Figure 4:**
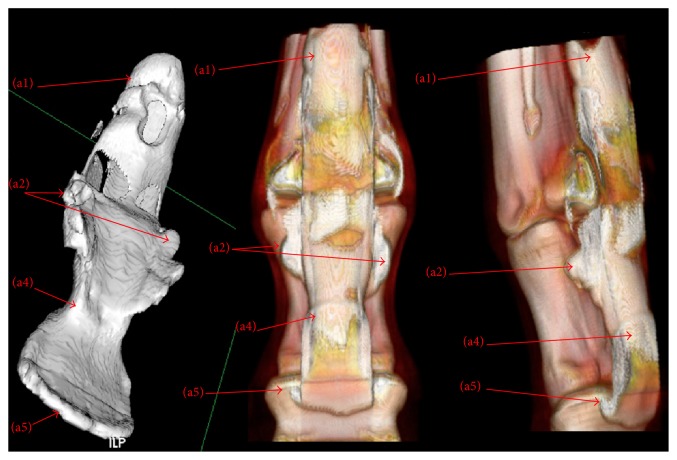
3D reconstruction. (1) Surface rendering; (2) volume rendering. (a1) Dorsal proximal recess, (a2) palmar proximal recess, (a3) collateral recess, (a4) palmar distal recess, and (a5) dorsal distal recess.

Figures 3 and 4 show representative images of MPR and 3D reconstructions of CT DFTS tenography. The image quality was considerably better using the overlapping scanning technique (pitch 0.6) with a retroreconstruction of a slice thickness < 1 mm.

### 3.3. Observer Scores of the Images

Table 1 shows the median scores of all the observers' scores for each structure.

A comparison of the scores assigned to the structures and artefacts of each specimen showed that the MF and synovial collateral recess were more easily recognized (*p* < 0.05) by the expert observers (score median 2) than the students (score median 1.5). All the other structures were easily recognized by both the experts and students.

Comparison of the scores assigned to each specimen showed that observers had more difficulty (*p* < 0.05) identifying the SDFT, DDFT, and collateral recess of specimen 6 than for the other specimens.

Comparison of the artefact scores of each specimen showed that the expert observers assigned a significantly lower median score (*p* < 0.05) to the specimen that was insufficiently distended by CM than the students. Specimen 6 had a significantly higher score (*p* < 0.05) than the other specimens because of insufficient distension by CM.

## 4. Discussion

To the best of our knowledge, this is one of the first study reports to describe the normal CT tenographic anatomy of DFTS in the horse.

The results of this study indicate that the CT tenography of normal horse forelegs allowed easy identification of the entire DFTS and intrathecal digital tendons. In particular, the MF was identified in all the specimens that were investigated; the mesotendons and synovial recess were also identified.

CT tenographic images of the DFTS were acquired rapidly with high-quality images. Both images obtained with submillimetric thickness (obtained with retroreconstruction) and overlapping technique were very useful in reconstruction for MPR and 3D images, allowing improvement of quality, especially for images obtained in the sagittal and parasagittal planes, which were fundamental to visualizing normal tendon structures in this study. In fact, with the standard contiguous scanning technique (slice thickness = 3 mm), reconstruction of images in planes other than transverse image quality was very low. The use of novel 4- to 16-slice CT scanner will provide submillimetric thickness slice, resulting in improvement of the image quality, even in MPR and 3D, in order to diagnose very small defects, avoiding partial volume artefacts. Multislice CT scanners could improve also scanning time resulting in lower anaesthesia time. Anaesthesia is required to perform in vivo CT scanning of the horse; a shorter CT procedure could prevent intra- and postanaesthesia complications and reduce associated costs. Notwithstanding in this study we employed a single-slice CT scanner, the scanning procedure was performed in less than 10 min for each specimen.

The concentration of CM (185 mg/mL) was sufficient for visualizing intrathecal structures; the total volume should be larger than 30 mL but should not exceed 60 mL, in order to obtain adequate synovial distension while avoiding CM leakage from the puncture site. This artefact did not affect the anatomical assessments performed on transverse scans. Adequate synovial distension has been reported to improve the image quality of the equine stifle joint [9].

Analysis of score assignments showed that the main structures were easily identified by both expert and student observers, apart from the synovial collateral recess and MF. These two structures were recognized more easily by the experts, probably because they are normally thin and require a skilled observer for accurate identification.

Artefacts (air bubbles, CM leakage, and reinforced beam) were similarly recognised and scored by both observer categories, not influencing image interpretation. Inadequate distension was found to be the main factor that affected the interpretation of images. This was illustrated by the structure scores of specimen 6 compared with the other specimens. Injecting half volume (30 mL) of CM, SDFT, and DDFT at the intrathecal level and also collateral recess were distinguished more difficultly (*p* < 0.05) by observers. We therefore can speculate that a total CM volume of 60 mL was adequate for optimal filling of synovial cavity allowing proper separation of structures and distension of recess, helping in image interpretation.

In conclusion, CT tenography of equine DFTS has been evaluated as an alternative diagnostic technique to visualize intrathecal structures digital tendons in the horse. This imaging method could be employed with adequate technique and good knowledge of the normal tomographic anatomy to test specificity and accuracy in diagnosing specific conditions affecting the digital sheath, mesotendon,* manica flexoria* or SDFT, and DDFT.

## Figures and Tables

**Table 1:** Median of total scores assigned by observers for normal structures and artefacts for each specimen.

Specimen number	1	2	3	4	5	6

Median total score of structures	32	31	32	30	32	29

Median total score of artefacts	5	4	5	4	6	6
